# Experimental investigation and prediction of the flexural properties of FDM printed carbon fiber reinforced polyamide parts using optimized RSM and ANN models

**DOI:** 10.1371/journal.pone.0322628

**Published:** 2025-05-19

**Authors:** Abdulsalam A. Al-Tamimi, Kenan Muhamedagic, Derzija Begic – Hajdarevic, Ajdin Vatres, Edin Kadric

**Affiliations:** 1 Department of Industrial Engineering, College of Engineering, King Saud University, Riyadh, Saudi Arabia; 2 Faculty of Mechanical Engineering, University of Sarajevo, Sarajevo, Bosnia and Herzegovina; Koneru Lakshmaiah Education Foundation / Indian and Xidian University, INDIA

## Abstract

The application of additive manufacturing technologies for producing parts from polymer composite materials has gained significant attention due to the ability to create fully functional components that leverage the advantages of both polymer matrices and fiber reinforcements while maintaining the benefits of additive technology. Polymer composites are among the most advanced and widely used composite materials, offering high strength and stiffness with low mass and variable resistance to different media. This study aims to experimentally investigate the impact of selected process parameters, namely, wall thickness, raster angle, printing temperature, and build plate temperature, on the flexural properties of carbon fiber reinforced polyamide (CFrPA) fused deposition modeling (FDM) printed samples, as per ISO 178 standards. Additionally, regression and artificial neural network (ANN) models have been developed to predict these flexural properties. ANN models are developed for both normal and augmented inputs, with the architecture and hyperparameters optimized using random search technique. Response surface methodology (RSM), which is based on face centered composite design, is employed to analyze the effects of process parameters. The RSM results indicate that the raster angle and build plate temperature have the greatest impact on the flexural properties, resulting in an increase of 51% in the flexural modulus. The performance metrics of the optimized RSM and ANN models, characterized by low MSE, RMSE, MAE, and MAPE values and high R^2^ values, suggest that these models provide highly accurate and reliable predictions of flexural strength and modulus for the CFrPA material. The study revealed that ANN models with augmented inputs outperform both RSM models and ANN models with normal inputs in predicting these properties.

## 1. Introduction

Additive manufacturing (AM) is a rapidly developing process in the manufacturing sector due to its ability to manufacture complex structures without the need for tooling and its ability to reduce waste in manufacturing. This is achieved by fabricating the product in a layer-by-layer fashion, which allows a great deal of control over the process and part details. Fused deposition modelling (FDM) process, a material extrusion AM technique, is one of the most used AM processes in industry and academia today because of its easy accessibility, low cost, ability to fabricate complex parts, appropriate part functionality, and rapid production [1–3]. Among the materials used in the FDM process, thermoplastic polymers, such as polyamide (PA), polylactic acid (PLA), acrylonitrile butadiene styrene (ABS), and polyethylene terephthalate glycol (PETG), are the most utilized materials [4]. Owing to the technological advancements in the AM FDM process and the range of materials, standalone materials have become insufficient for high-load bearing applications. For example, research has been conducted to improve the mechanical properties of FDM printed parts made of pure thermoplastic polymers by using carbon fiber reinforcement [5,6]. Compared with those of pure PLA, the tensile strength and flexural strength of FDM printed parts made of carbon fiber reinforced PLA are almost three times greater [5]. Additionally, the tensile strength of polyamide 6 (PA6) reinforced with 25% carbon fibers was more than three times greater than the tensile strength of pure PA6 [6]. As one of the most important composites for the FDM process, carbon fiber-reinforced polyamide has been widely used in many fields due to its excellent properties [7]. Peng et al. [8] investigated the effects of printing orientation and build plate temperature on the tensile strength and interlayer adhesion of samples fabricated from PA6 reinforced with carbon fibers. In addition, the impact of short carbon fibers on the electrical, mechanical and piezoresistivity properties of FDM printed PA composite parts was studied [9]. The results showed that the presence of carbon fibers improved the mechanical properties of the PA composite compared with those of pure PA. However, the presence of carbon fibers reduced the impact strength, whereas the fracture toughness of the PA composite was slightly greater than that of pure PA. Su et al. [10] analyzed the influence of the carbon fiber content on the tensile and thermal properties, fiber length, fiber orientation and porosity of the FDM printed PA composite samples. Compared with those of the unreinforced PA samples, the tensile strength and modulus of the FDM-printed PA samples reinforced with 20% carbon fibers were significantly greater. However, the mechanical performance of samples with 40% carbon fiber reinforcement was significantly reduced as a result of poor fiber alignment and high porosity. Matsika Klossa et al. [11] found that samples of nylon reinforced with 10% carbon fiber exhibit greater tensile mechanical properties than samples with 0%, 5% and 20% carbon fiber reinforcement. The effect of infill density on the thermal and mechanical properties of carbon fiber reinforced polyamide composites fabricated by FDM process was studied previously [12]. The effects of infill density and infill patterns on the mechanical properties of FDM fabricated parts of carbon fiber reinforced polyamide 6 were investigated in [13], and the effects of these two process parameters on the tensile properties of reclaimed carbon fiber reinforced PA6 composites were analyzed [14]. Wang et al. [15] studied the effects of layer thickness and nozzle temperature on the tribological and mechanical properties of FDM printed polyamide parts. Shashikumar et al. [16] investigated the structural, mechanical, and morphological properties of PA6 reinforced with 20% short carbon fibers. The results revealed that the samples with a ± 45° raster orientation presented the highest tensile strength compared with those with raster orientations of 0°, 45° and 90°, whereas the highest impact energy was achieved in the samples with a 90° raster orientation. Zeybek et al. [17] investigated the effect of strain rate on the compressive properties of short carbon fiber reinforced PA6 composites and neat PA6 and reported that the PA composite exhibited increased compression stress. A review of the literature indicates that process parameters significantly impact the mechanical performance of FDM-printed parts, and various studies have been conducted to enhance this performance [18,19]. However, not all process parameters affect every mechanical property uniformly, and their effectiveness can vary depending on the material type and mechanical properties. Muhamedagic & Cekic [20] explored the flexural properties of carbon fiber-reinforced polyamide, focusing on parameter optimization via the Taguchi method. However, the study offers only a preliminary investigation and lacks predictive modeling or a detailed analysis of the composite’s behavior under flexural loads, restricting its applicability for broader material design considerations. This highlights a gap in the literature regarding investigations of the effects of FDM process parameters on the flexural properties of PA6-CF composites.

The selection of optimal process parameters is crucial for improving the mechanical properties of FDM printed components. Researchers have applied different techniques, such as response surface methodology (RSM), Taguchi design, particle swarm optimization (PSO), gray relational analysis (GRA), artificial neural networks (ANNs), and genetic algorithms (GAs), to find the optimal process parameters to improve the mechanical properties [5,21,22]. The RSM method offers the possibility to obtain, based on face-centered central composite design, an extensive amount of data that can be used for the analysis of a certain process. Additionally, RSM is used for the prediction of output variables and the selection of input parameters. On the other hand, ANNs are suitable tools for modeling and evaluating complex problems that exhibit a nonlinear relationship between factors and responses. Giri et al. [23] investigated the use of ANNs to optimize the effects of the layer thickness, raster angle, raster width, air gap, build orientation, and number of contours on the tensile strength, surface roughness, and build time of FDM printed PLA parts. Joy et al. [24] developed a model using an ANN to predict the tensile strength of FDM parts fabricated from carbon fiber reinforced ABS. Deshwal et al. [25] applied an RSM based on central composite design (CCD) to develop a model for tensile strength. Furthermore, hybrid models such as GA-ANN, GA-RSM and GA-ANFIS were used to optimize the process parameters (infill density, temperature and speed). RSM and an ANN were applied in [26] to predict the tensile strength of FDM parts made of carbon fiber reinforced PAs. Saad et al. [27] applied RSM based on CCD design to analyze the effects of different parameters, such as printing speed, layer thickness, outer shell speed, and print temperature, on the flexural strength of FDM printed PLA parts. Furthermore, a PSO was applied for the optimization of process parameters to improve the flexural strength of parts. Selvamani et al. [28] used RSM to predict the bending and compression properties of FDM parts made with 15% and 70% brass reinforced PLA. The study found that samples with 15% brass composition exhibited better properties than did samples with 70% brass. RSM based on the Box–Behnken design was used in [29] to investigate the effects of process parameters such as infill density, print angle and perimeter count on the tensile strength of FDM printed PLA parts. Mishra et al. [30] developed a finite element model to predict the flexural and tensile strengths of FDM printed PA parts. The proposed model was confirmed by experiments, and the average prediction accuracy was 95% for the tensile samples and 87% for the flexural samples.

Estimating parameter effects using traditional regression methods such as RSM is constrained in modeling highly nonlinear relationships between process parameters and mechanical properties. However, the key advantage of these statistical models is that they are easy to understand and interpret. In contrast, ANNs can achieve superior predictive performance but lack interpretability. ANN performance is highly dependent on predefined hyperparameters, which are typically determined through labor-intensive trial-and-error procedures, resulting in varying outcomes. A more systematic approach involves the application of hyperparameter optimization techniques, such as grid search or Bayesian optimization [31,32]. Another well-known limitation of ANNs is the requirement of large number of samples for effective training. This limitation is particularly pronounced in material research, where Design of Experiments (DOE) techniques are commonly applied to minimize the time and resource requirements for samples production [24,25,33].

This study addresses the abovementioned research gaps by investigating the effects of FDM process parameters on the flexural properties of 3D printed CFrPA FDM. To the best of the authors’ knowledge, this is the first study to develop a prediction model for the flexural behavior of CFrPA composites. Given the superior properties of CFrPA composites, which make them ideal for industrial application in automotive parts that require high performance at elevated temperatures, it is crucial to explore how process parameters influence the flexural strength of these parts. Flexural samples were fabricated using the FDM additive manufacturing technique and tested in accordance with ISO 178 standards. The influences of key FDM process parameters, including wall thickness, raster angle, printing temperature, and build plate temperature, on flexural properties were systematically analyzed and discussed. By employing advanced statistical techniques for optimal process parameter selection, the flexural properties of FDM-printed parts can be enhanced. Prediction models were developed using RSM and ANN, followed by a comparative analysis of their performance. Two types of ANN models were developed, one using normal (standard) and the other augmented inputs. Shapley Additive Explanations (SHAP) was employed to analyze and explain ANN predictions. Furthermore, this study discusses ANN hyperparameter selection and optimization. Rather than relying on trial-and-error methods, a random search technique was implemented to optimize key ANN hyperparameters. To ensure model generalizability and prevent overfitting, particularly given the relatively small dataset, a five-fold cross-validation procedure was employed.

The remainder of this paper is organized as follows: Section 2 outlines the methodology, including specimen design and printing parameter selection, mechanical property testing procedures, experimental design using DSD, and analysis of variance. Section 3 defines the ANN structure, including data preprocessing, the ANN architecture and hyperparameter optimization, and performance metrics. Section 4 presents the main results of the study, including a detailed discussion of the predictions and the effects of the four printing parameters on the mechanical properties of the CFrPA material. Finally, Section 5 provides the main conclusions of the study.

## 2. Materials and methods

### 2.1. Design and 3D printing

**Fig 1:**
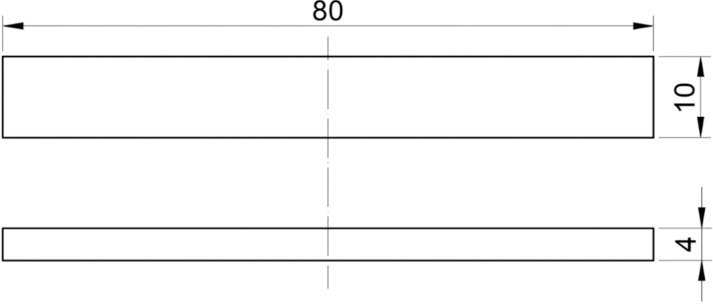
Test sample for flexural testing according to ISO 178.

**Fig 2:**
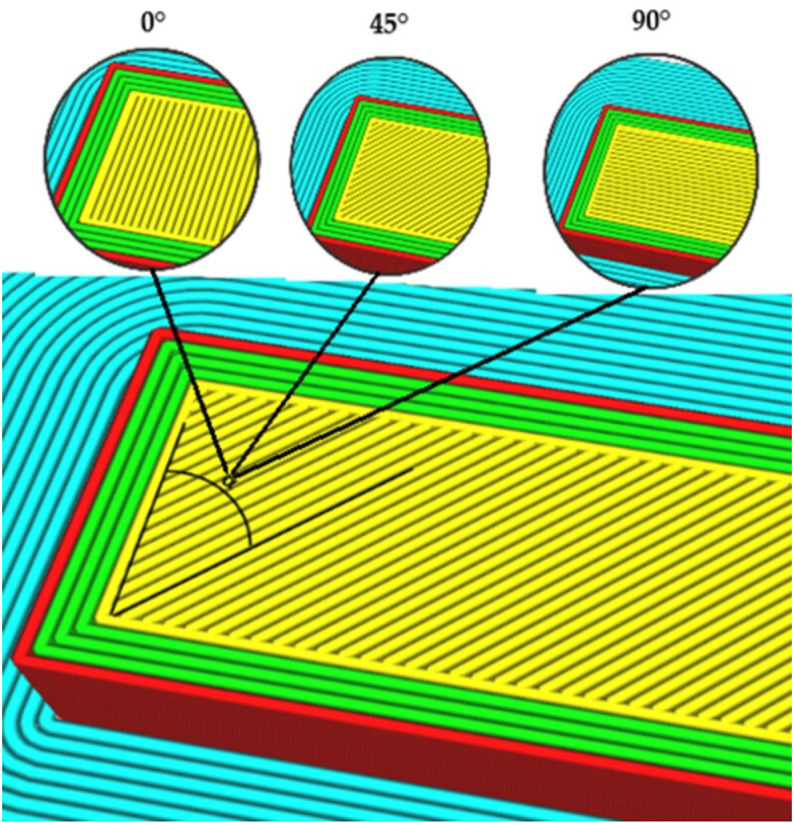
Material deposition patterns for different raster angles.

Flexural test samples were designed using Solidworks 2021 (Dassault Systemes, Velizy-Villacoublay, France) according to the ISO 178 standard, as shown in Fig 1. The CAD models were transferred to an UltiMaker Cura 5.4.0 slicer as a stl. file to prepare the model for 3D printing by slicing the models, determining the process parameters, and exporting it as a G-code file. The G-code file is then imported to the printer, and samples are printed using a material extrusion Ultimaker S3 (UltiMaker, Utrecht, The Netherlands). A polymer composite filament material of polyamide 6 reinforced with 10% short carbon fibers (CFrPA) was purchased from Royal DSM (Novamid® ID1030 CF10, Royal DSM, Heerlen, The Netherlands). To eliminate the impact of moisture, the filaments were dried at 80 °C for 6 hours before printing. The UltiMaker Print Core CC (0.6 mm) nozzle was acquired from UltiMaker (UltiMaker, Utrecht, The Netherlands). The selected nozzle has a diamond tip that is highly resistant to abrasion, which is crucial in the deposition of composite materials. On the basis of preliminary tests [20] four influential process parameters, namely, wall thickness (A), raster angle (B), printing temperature (C), and build plate temperature (D) were selected. Also, the results of preliminary tests showed that layer thickness and printing speed were not statistically significant parameters, and therefore these parameters were kept constant during the experiment. The raster angle and wall thickness are the structural parameters that define the pattern of material deposition (Fig 2). These four selected process parameters are varied at three levels (low, middle, and high), as shown in Table 1. The other process parameters were kept constant and are presented in Table 2.

**Table 1:** Process parameters and their levels.

Process parameters	Symbol	Unit	Levels		
			Low	Middle	High
Wall thickness	A	mm	0.8	1.4	2
Raster angle	B	°	0	45	90
Printing temperature	C	°C	240	260	280
Build plate temperature	D	°C	50	90	130

**Table 2:** Constant parameters and their values.

Layer thickness	Printing speed	Infill	Part orientation	Nozzle diameter
0.1 mm	60 mm/s	100%	Flat x-x direction	0.6 mm

### 2.2. Flexural test

**Fig 3:**
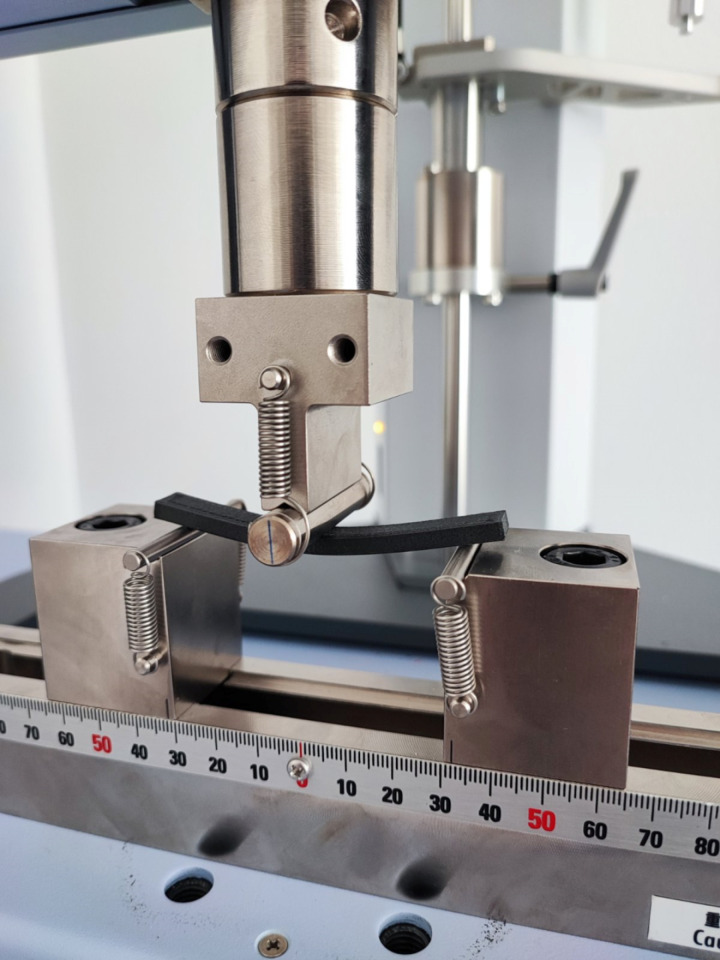
Flexural testing of the FDM printed samples.

Flexural testing was conducted following the ISO 178 standard at room temperature using a universal testing machine Shimadzu AGS-X universal testing machine (Shimadzu Corporation, Kyoto, Japan) with a maximum load cell of 10 kN, as shown in Fig 3. A 5 mm/min deflection rate with data sampling of 50 Hz was used. The flexural strength (SF) and flexural modulus (E) were calculated from the recorded stress–strain curves obtained in Shimadzu Trapezium-X software version 1.5.2 (Shimadzu Corporation, Kyoto, Japan). Testing was performed until failure criteria were achieved, as defined by significant deformation, cracking, or breakage of the sample. The measured responses were then imported into Design Expert version 13 software (Stat-Ease, Inc., Minneapolis, MN, USA) for further statistical analysis. The flexural tests were replicated three times for each sample.

### 2.3. Design of experiment using RSM

To develop regression models for predicting and analyzing the flexural properties of FDM printed samples, an RSM based on a face-centered central composite design (FCCD) was used. The experimental data for 30 runs with four process parameters and the results of the two responses, namely, the flexural strength (SF) and flexural modulus (E), are given in Table 3.

**Table 3:** Design matrix with process parameters and measured responses.

Exp. No.	Process parameters				Responses (average of three samples)	
	A	B	C	D	SF (MPa)	E (GPa)
1	1.4	45	260	90	133.80	4.64
2	0.8	90	240	130	182.13	6.49
3	1.4	0	260	90	121.57	4.06
4	2	0	280	130	141.80	4.86
5	0.8	0	280	130	117.61	3.64
6	2	45	260	90	141.04	5.13
7	2	0	240	50	97.56	4.31
8	1.4	45	240	90	120.54	4.31
9	0.8	0	240	130	110.62	3.59
10	2	0	280	50	121.73	4.49
11	0.8	45	260	90	118.69	3.96
12	1.4	90	260	90	170.49	6.63
13	0.8	90	240	50	144.19	6.00
14	2	90	240	130	180.70	7.01
15	2	90	240	50	148.70	6.31
16	0.8	0	280	50	107.90	3.59
17	0.8	0	240	50	77.40	3.43
18	1.4	45	260	90	130.20	4.43
19	1.4	45	260	130	134.82	4.53
20	2	90	280	50	153.60	6.31
21	2	0	240	130	132.22	4.70
22	1.4	45	260	90	136.30	4.75
23	1.4	45	260	90	135.60	4.65
24	0.8	90	280	130	185.04	6.67
25	0.8	90	280	50	150.27	6.12
26	1.4	45	280	90	127.31	4.26
27	1.4	45	260	90	137.83	4.80
28	1.4	45	260	90	130.20	4.50
29	1.4	45	260	50	112.28	4.06
30	2	90	280	130	183.00	6.63

The results of the flexural strength and modulus tests were collected and used to create a representative mathematical model. First, different mathematical models for predicting flexural properties were evaluated, and then, adequate models were selected. Analysis of the experimental data and development of regression models for the prediction of SF and E are performed using Design Expert version 13 software (Stat-Ease, Inc., Minneapolis, MN, USA). All the statistical tests were performed at the 0.05 significance level. The analysis of variance (ANOVA) was used to assess the statistical significance of the process parameters based on the F values and their corresponding p-values. Process parameters with p-values less than 0.05 were considered statistically significant. Regression analysis is used to fit the experimental data to a quadratic regression model, given by:


$$y=\beta_{0}+\sum_{i=1}^{k}\beta_{i}x_{i}+\sum_{i=1}^{k}\beta_{ii}x_{i}^{2}+\sum_{i=1}^{k-1}\sum_{j>i}^{k}\beta_{ij}x_{i}x_{j}+\epsilon$$ (1)

where *y* is the response; $x_{i}$ and $x_{j}$ are factors; $\beta_{0}$ is the intercept; $\beta_{i}$, $\beta_{ii}$ and $\beta_{ij}$ are coefficients of linear, quadratic and two-factor interaction terms, respectively; k is the number of factors; and ϵ is the random normally distributed error.

## 3. Artificial neural network

### 3.1. Structure of ANN

An artificial neural network (ANN) is a computational model inspired by a simplified representation of biological brain structure and function and is composed of interconnected nodes organized into layers [34]. Neural networks are considered universal function approximators [35–38] and can generally be used to approximate any function with an arbitrary degree of accuracy. ANNs can discover and learn hidden relationships and complex patterns within data and are far more general, flexible and representationally powerful than traditional regression models. Simpler ANNs represent the extension and generalization of regression models.

**Fig 4:**
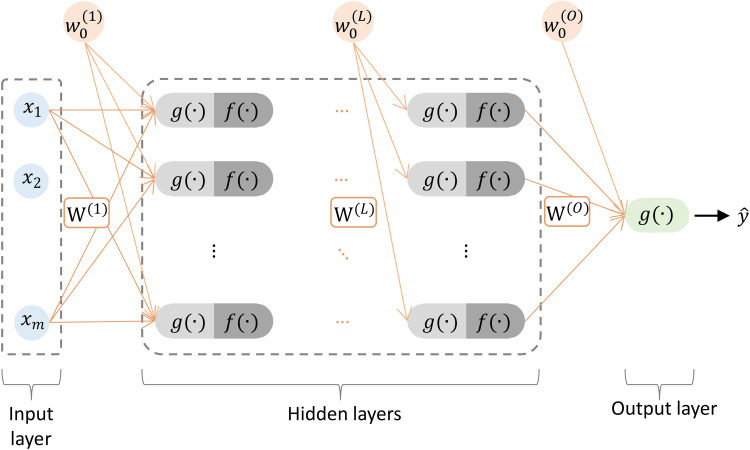
Conventional MLP-ANN architecture.

Owing to their advantageous properties, ANNs have been applied in various fields [39], especially in engineering and materials science [31–33,40]. In the context of modeling 3D printed material mechanical properties, ANNs are well suited for modeling complex relationships between inputs (printing parameters) and outputs (material properties). Among various ANN models, the most popular are multilayer perceptron neural networks (MLP-ANN), which belong to the class of feedforward neural networks. These models are relatively simple, with simple architecture and relatively small numbers of parameters (weights). The conventional MLP-ANN architecture includes an input layer, one or more hidden layers, and an output layer, with each node in a layer connected to every node in the subsequent layer, as shown in Fig 4.

In the conventional neural network architecture, data flow in a forward direction from the input to the output layer, and in each layer, a specific set of transformations is applied to the input data. Each neuron performs two operations. First, the input data vector $X\;=\;\left[x_{1},\;x_{2},\;\ldots ,\;x_{m}\right]$ from the preceding layer is multiplied by the weight vector $W\;=\;\left[w_{1},\;w_{2},\;\ldots ,\;w_{m}\right]$, to which a fixed value $w_{0}$, also known as bias, is added. This operation can be mathematically defined as:


$$g(x)=\sum_{i=1}^{m}w_{i}^{\left(l\right)}x_{i}+w_{0}^{\left(l\right)}$$ (2)

Second, the output of g is then passed to an activation function f, which additionally transforms g. The activation function f is a simple, fixed, and differentiable nonlinear function. There are many types of activation functions, however, the commonly used functions are tanh, sigmoid and relu, defined as follows:


$$\tanh(x)=\frac{e^{x}-e^{-x}}{e^{x}+e^{-x}}$$

$$sigmoid(x)=\;\frac{1}{1+e^{-x}}$$

relu(x)=max(0,x) (3)

Vector of f’s in each layer can now be considered as input to the subsequent layer. The complete transformation of inputs to outputs in an ANN can be mathematically defined as a sequence of function compositions as follows:


$$\hat{y}=\left(g_{L}\circ \ldots \circ f_{l}\circ \;g_{l}\circ \ldots \circ f_{1}\circ g_{1}\right)(X)$$ (4)

where $\hat{y}$ is the output of the ANN, X is the input to the ANN, $g_{l}$ is the weighted sum function, $f_{l}$ is the activation function, and L is the number of hidden layers in ANN (1≤l≤L).

The learning process of ANNs involves iterative updates of the weights and biases of the network by optimization algorithms, in which a predefined loss function is minimized. This iterative update of weights and biases is performed in the backward pass via backpropagation and gradient descent algorithms. However, the learning process and performance of the ANN are highly influenced by a set of predefined hyperparameters, including the network architecture, which is defined by the number of layers, the number of neurons in each layer, the types of layers’ activation functions, the batch size, and the learning and decay rates. The batch size represents the number of training samples simultaneously fed into the network in the forward pass, for which the average gradients are computed in the backward pass. The learning rate is used in the optimization algorithm to control the rate of weight update in the backward pass. The decay rate is used in the optimization algorithm to reduce the likelihood of overfitting by penalizing large weight values. To ensure the high prediction performance of the ANN, it is essential to optimize the network in terms of its hyperparameter values.

### 3.2. Data preprocessing for use in ANN

The experimental matrix (Table 3) with 30 experimental runs, among which 6 are central point runs, is reduced to prevent cases where central point runs are simultaneously present in the training and test datasets. All central point runs (6 runs where all factors are at their middle levels) are removed and replaced with one run with average values of each of the responses. The new reduced experimental matrix, with 25 runs, is then used for hyperparameter optimization, training and validation of the ANN. To enhance network training convergence, the natural values of the process parameters and responses are normalized into ranges of [-1, 1] and [0, 1], respectively.

Two approaches for feeding data into the network are considered, namely, normal and augmented inputs, which differ in their input sizes. In the first approach, inputs to the ANN are given in columns A, B, C, and D of the experimental matrix (Table 3), which represent process parameters. In the second approach, the experimental matrix is augmented by adding columns representing two-factor interactions and quadratic terms (AB, AC, …, DD). The reason for these two approaches is based on the expected nonlinear relationships between responses and factors. While ANN is capable of approximating nonlinear relationships between inputs and outputs, it is reasonable to expect that ANN with normal inputs will be more complex, as it will need more layers, neurons, and parameters to ensure nonlinear mappings of inputs into outputs. Conversely, an ANN with augmented input, which includes interaction and quadratic terms, is expected to have a simpler structure and fewer parameters and, eventually, will not need to provide nonlinear but rather linear mappings.

### 3.3. ANN architecture and hyperparameter optimization

To determine the “optimal” architecture and hyperparameter values of an ANN model and consequently select the best models for the prediction of flexural strength (SF) and flexural modulus (E), several configurations with various architectures and hyperparameters are trained and validated. The hyperparameter search space for configurations with normal and augmented inputs is given in Table 4. Assuming that models with augmented input should have simpler architecture than models with normal input, the number of layers is reduced from 3 to 2, and the number of neurons in layers is also changed.

**Table 4:** Hyperparameter search space for ANN with normal and augmented inputs.

Hyperparameter	ANN with normal input	ANN with augmented input
Number of layers	(1-3)	(1-2)
Number of neurons	([4 - 14], [2 - 6], [2 - 10])	([1 - 14], [2 - 8])
Activation function	(linear, tanh, sigmoid, relu)	(linear, tanh, sigmoid, relu)
Batch size	(1, 5)	(1, 5)
Learning rate	(0.001, 0.0001)	(0.001, 0.0001)
Decay rate	(0.001, 0.0001)	(0.001, 0.0001)

**Fig 5:**
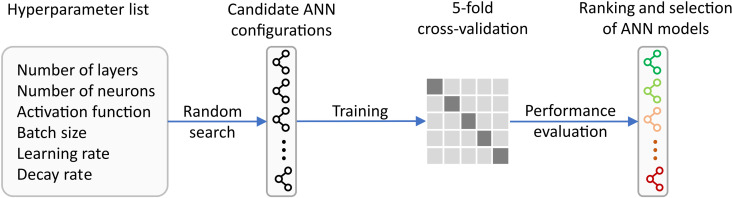
Procedure for ANN hyperparameter optimization and selection of the best performing models.

The procedure used to determine the best performing ANN models with normal and augmented inputs for the prediction of SF and E is presented in Fig 5. Considering the hyperparameter search space, there are 17952 and 3584 unique ANN configurations for normal and augmented inputs, respectively. Due to the high computational cost of evaluating all configurations, a random search approach is used to generate 100 ANN models, which are then trained and validated on the same dataset (experimental matrix). This approach ensures sufficient and unbiased random model selection and consequently achieves generalization of ANN model performance in predicting SF and E. The five-fold cross-validation is used to train and validate each model by splitting the dataset five times into training and validation datasets over 1000 epochs at a ratio of 80/20, respectively. The mean squared error (MSE) is used as the loss function in the training and validation of ANN models. The final performances of the models are estimated as the average of the five-fold MSEs. Finally, the candidate models are ranked by average MSE such that the model with the lowest average MSE is the best (first ranked). These models are selected for the prediction of SF and E.

### 3.4. SHAP analysis

Artificial neural networks are commonly referred to as black-box models due to their complex architecture with long sequence of nonlinear transformations that map input data to outputs, lacking a clear and concise mathematical representation of the relationships between inputs and outputs. This lack of clearly defined analytical relationships presents challenges in understanding the underlying processes and the impact of input data on model outputs. To address this well-known and limiting property of machine learning models, various methodologies are developed with focus on explaining and interpreting such models [41].

In recent years, concepts from coalitional game theory have been successfully applied to machine learning model interpretation, particularly in SHAP method. This method treats a model’s predictive performance and input features as a payoff that can be freely distributed among players [42].

In the interpretation and explanation of complex models, such as an artificial neural network f(x) with input features $x=(x_{1},x_{2},\ldots ,x_{M})$, the SHAP method proposes the use of a simpler explanation model. Inputs of this explanation model are binary variables $z^{\prime }$ referred to as simplified features. Under the assumption that there exists mapping from simplified to input features $h_{x}\left(z^{\prime }\right)=x$, the explanation model can be defined in the form of a linear function of binary variables, $g(z^{\prime })$:


$$g\left(z^{\prime }\right)=\phi_{0}+\sum_{i=1}^{M}\phi_{i}z_{i}^{\prime },$$ (5)

where $z^{\prime }\in {\left\{0,1\right\}}^{M}$, M is the number of input features, and $\phi_{i}$ is the effect attributed to each feature i known as the SHAP value of feature i [42].

Equation (5) has a unique solution with three properties essential for interpretability: local accuracy, missingness and consistency. Local accuracy ensures sensible explanation of any instance x. Namely the explanation model g will match the output of the original model f for the simplified input $z^{\prime }$, corresponding to the original instance x. Missingness implies that feature i, not present in the original input x, has no impact on the explanation model g and should have an attribution $\phi_{i}=0$. Consistency means that if a model f is modified to increase the effect of a feature i on the model’s output, while the effects of all other features remain constant, the corresponding attribution $\phi_{i}$ should increase or stay the same [42].

### 3.5. Performance metrics

The prediction accuracy of the RSM and ANN models is assessed through performance metrics such as the mean squared error (MAE), root mean squared error (RMSE), mean absolute error (MAE), mean absolute percentage error (MAPE) and coefficient of determination ($R^{2}$). The MSE, RMSE, MAE and MAPE are used to estimate the deviations of the model predictions from the experimental data. Smaller values of these metrics are preferable, indicating better agreement between the experimental data and model predictions. $R^{2}$ is used to estimate correlations between experimental data and model predictions, with a value of $R^{2}$ equal to 1 indicating perfect agreement between them.

The performance metrics are calculated using the following equations:


$$MSE\;=\frac{1}{n}\sum_{i=1}^{n}{\left(y_{i}-{\hat{y}}_{i}\right)}^{2}$$

$$RMSE=\sqrt{MSE}$$

$$MAE\;=\frac{1}{n}\sum_{i=1}^{n}\left|y_{i}-{\hat{y}}_{i}\right|$$

$$MAPE=\frac{1}{n}\sum_{i=1}^{n}\left|\frac{y_{i}-{\hat{y}}_{i}}{y_{i}}\right|100\%$$

$$R^{2}=1-\frac{\sum_{i=1}^{n}{\left(y_{i}-{\hat{y}}_{i}\right)}^{2}}{\sum_{i=1}^{n}{\left(y_{i}-\overset{―}{y}\right)}^{2}}=\frac{\sum_{i=1}^{n}{\left({\hat{y}}_{i}-\overset{―}{y}\right)}^{2}}{\sum_{i=1}^{n}{\left(y_{i}-\overset{―}{y}\right)}^{2}}$$ (6)

where $y_{i}$ and ${\hat{y}}_{i}$ are experimental and predicted values, respectively, $\overset{―}{y}$ is the average experimental value, and n is the total number of observed values in the dataset.

## 4. Results and discussion

### 4.1. Prediction of Flexural Properties by RSM

**Fig 6:**
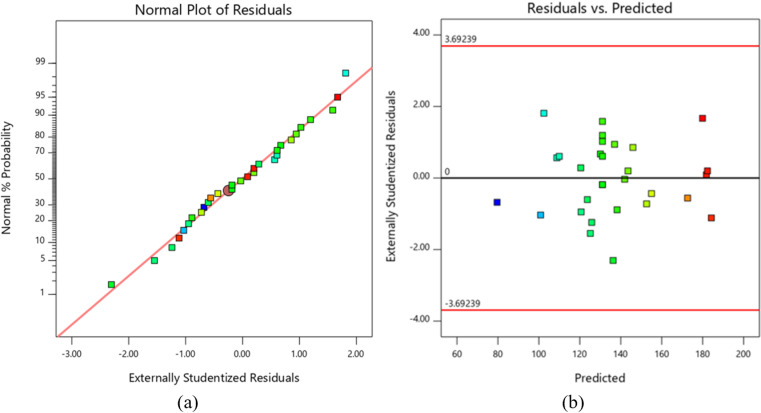
(a) Normal probability plot of residuals and (b) residuals vs. predicted plot of the model for predicting SF.


Table 5 presents the model summary statistics, with the recommended model for predicting flexural strength. Based on the ANOVA results, a quadratic model for predicting SF was proposed. After removing the insignificant terms (p > 0.05), a reduced quadratic model for predicting SF was developed. The ANOVA results for this reduced quadratic model are shown in Table 6. The p-value of 0.0001 indicates the model’s significance, with only a 0.01% probability that such a large F-value could result from noise. The lack-of-fit p-value of 0.1574 (p > 0.05) suggests that the deviation from the model is not statistically significant. The obtained values of R^2^, Adjusted R^2^, and Predicted R^2^ for the flexural strength of the FDM-printed parts confirm the adequacy of the reduced quadratic model. These coefficients demonstrate a strong relationship between the model and the process parameters. The normal probability plot of the residuals and the residuals versus the predicted plot of the model for predicting SF, as presented in Fig 6, shows high agreement between the experimental and model predicted data. The residuals are normally distributed, and the variance is constant. The reduced quadratic model for predicting flexural strength is given by:

**Table 5:** Model summary for SF.

Source	Lack of Fit *p*-value	R^2^	R^2^_adj_	R^2^_pred_	Remarks
Linear	0.0089	0.8890	0.8712	0.8334	
2FI	0.0116	0.9283	0.8905	0.8136	
Quadratic	0.1270	0.9818	0.9647	0.9128	Suggested
Cubic	0.0697	0.9922	0.9676	0.3583	Aliased
**Reduced Quadratic**	**0.1574**	**0.9783**	**0.9669**	**0.9498**	**Selected**

**Table 6:** ANOVA of the reduced quadratic model for predicting SF.

Source	Sum of Squares	df	Mean Square	Contribution	*F*-value	*p*-value	Remarks
Model	18679.14	10	1867.91		85.74	< 0.0001	sig.
A	630.13	1	630.13	3.30	28.92	< 0.0001	
B	12257.08	1	12257.08	64.19	562.63	< 0.0001	
C	492.98	1	492.98	2.58	22.63	0.0001	
D	3592.98	1	3592.98	18.82	164.93	< 0.0001	
AB	355.42	1	355.42	1.86	16.31	0.0007	
BC	189.41	1	189.41	0.99	8.69	0.0082	
BD	83.04	1	83.04	0.43	3.81	0.0658	
CD	120.29	1	120.29	0.63	5.52	0.0297	
B^2^	828.75	1	828.75	4.34	38.04	< 0.0001	
D^2^	167.26	1	167.26	0.88	7.68	0.0122	
Residual	413.92	19	21.79	2.16			
Lack of Fit	362.49	14	25.89		2.52	0.1574	not sig.
Pure Error	51.44	5	10.29				
Cor Total	19093.07	29					
Std. Dev.	4.67					R^2^	0.9783
Mean	136.17					R^2^_adj_	0.9669
C.V. %	3.43					R^2pred^	0.9498

A – wall thickness, B – raster angle, C – printing temperature, D – build plate temperature.

SF=−159,24335+17,71632A+1,01493B+0,742159C+1,97133D

−0,17456AB−0,003823BC+0,001266BD−0,003427CD

$$+0,00766B^{2}-0,004355D^{2}$$ (7)

**Fig 7:**
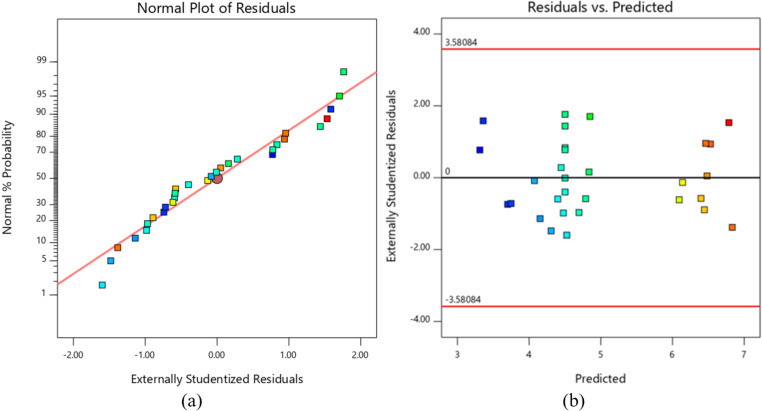
(a) Normal probability plot of residuals and (b) residuals vs. predicted plot of the model for predicting E.

Using a similar approach to the selection of the model for predicting SF, various regression models for predicting the flexural modulus were evaluated, as shown in Table 7, and an adequate model was then selected. A reduced quadratic model for predicting E was developed, and the ANOVA results are presented in Table 8. The p-value of < 0.0001 and the nonsignificant lack of fit (p = 0.2495) indicate that the flexural modulus is highly dependent on the input parameters. Additionally, the obtained values of R^2^ = 97.71, R^2^_adj_ = 97.11, and R^2^_pred_ = 96.30 for the flexural modulus of the FDM-printed parts confirm the adequacy of the selected model. The normal probability plot of the residuals and the residuals versus the predicted value for the flexural modulus are shown in Fig 7. The plots reveal that the residuals are closely clustered along a straight line, further indicating the model’s accuracy.

**Table 7:** Model summary for E.

Source	Lack of Fit *p*-value	R^2^	R^2^_adj_	R^2^_pred_	Remarks
Linear	0.0043	0.8352	0.8088	0.7644	
2FI	0.0028	0.8574	0.7824	0.5589	
Quadratic	0.2599	0.9862	0.9734	0.9443	Suggested
Cubic	0.1073	0.9928	0.9702	0.4255	Aliased
**Reduced Quadratic**	**0.2495**	**0.9771**	**0.9711**	**0.9630**	**Selected**

**Table 8:** ANOVA of the reduced quadratic model for predicting E.

Source	Sum of Squares	df	Mean Square	Contribution	*F*-value	*p*-value	Remarks
Model	33.40	6	5.57		163.29	< 0.0001	sig.
A	2.18	1	2.18	6.38	63.87	< 0.0001	
B	25.68	1	25.68	75.13	753.35	< 0.0001	
C	0.0098	1	0.0098	0.028	0.2875	0.5970	
D	0.6806	1	0.6806	1.99	19.96	0.0002	
AB	0.6123	1	0.6123	1.79	17.96	0.0003	
B^2^	4.24	1	4.24	12.41	124.33	< 0.0001	
Residual	0.7840	23	0.0341	2.29			
Lack of Fit	0.6834	18	0.0380		1.89	0.2495	not sig.
Pure Error	0.1007	5	0.0201				
Cor Total	34.18	29					
Std. Dev.	0.1846					R^2^	0.9771
Mean	4.69					R^2^_adj_	0.9711
C.V. %	3.72					R^2^_pred_	0.9630

A – wall thickness, B – raster angle, C – printing temperature, D – build plate temperature.

The reduced quadratic model for predicting the flexural modulus is given by:


E=2,06567+0,905671A+0,002588B+0,001167C+0,004861D

$$-0,007245AB+0,000379B^{2}$$ (8)

### 4.2. Effect of process parameters on flexural properties

**Fig 8:**
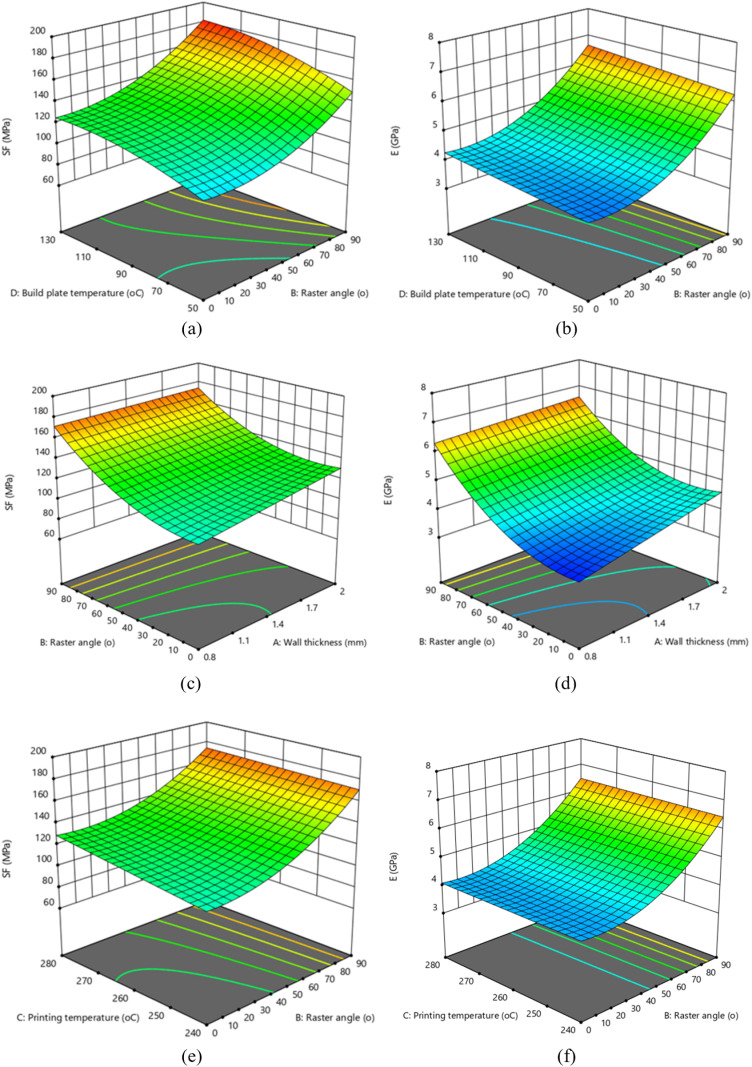
3D response surface plots of flexural properties: (a) SF vs. raster angle and built plate temperature; (b) E vs. raster angle and built plate temperature; (c) SF vs. raster angle and wall thickness; (d) E vs. raster angle and wall thickness; (e) SF vs. printing temperature and raster angle; (f) E vs. printing temperature and raster angle.

Fig 8 shows the surface plots of SF and E for all the considered process parameters. The results showed that the raster angle has the most significant effect, which agrees with the ANOVA. Raster angle determines the filament deposition angle of the infill pattern. It is one of the structural parameters and has a significant impact on the anisotropic behavior of a material with respect to the loading direction. Raster angle directly affects the direction of material deposition, thereby greatly influencing the mechanical properties of FDM manufactured parts. In this study, the flexural strength and flexural modulus are greater at 90^o^ raster angle and are directly proportional. SF increased by 139% from 77.4 MPa (exp. 17) to 185.04 MPa (exp. 24) when the raster angle was changed from 0^o^ to 90^o^. Also, E increased by 104% from 3.43 GPa (exp. 17) to 7.1 GPa (exp. 14) when the raster angle was changed from 0^o^ to 90^o^. The significant increase in the flexural properties of the CFrPA composite FDM printed samples at 90^o^ raster angle can be explained by the direction of material deposition and the direction of the carbon fibers. In a 90° raster orientation, the direction of raster deposition is parallel to the bending plane, and the carbon fibers are primarily aligned with the raster deposition. This alignment increases the resistance of the sample to bending. A similar effect of the raster angle on the flexural properties was also reported for ABS reinforced with short carbon fibers [43] and for the PLA material [44].

Additionally, the interaction between the raster angle and build plate temperature positively correlates with the flexural strength (Fig 8a) and flexural modulus (Fig 8b). An increase in flexural properties with increasing build plate temperature can be explained by a better cohesive bond between individual layers. At a higher build plate temperature, better heating of the previously deposited filament layers is enabled, which contributes to the reduction in the temperature gradient between the previously deposited layer and the currently deposited layer. By reducing the temperature gradient, the internal stresses are also reduced, and a stronger interlayer bond is achieved. If the interlayer bond is weak, shrinkage of the currently deposited layer may cause it to separate from the previously deposited layer, resulting in interlayer failure and a decrease in flexural properties. Moreover, the printing temperature and wall thickness had a tertiary influence on the flexural properties compared with the other two parameters considered (Fig 8c–8f). The maximum values of SF and E were obtained at different combinations of printing temperatures and wall thicknesses. This behavior can be caused by ineffective interraster bonding or the presence of interraster and in-raster failures.

**Fig 9:**
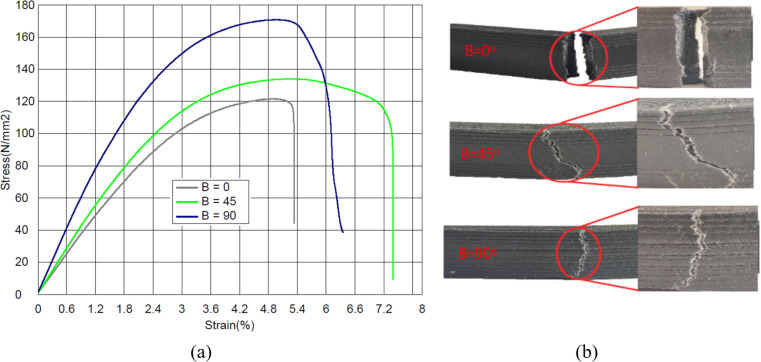
(a) Stress–strain curves at raster angles of 0^o^, 45^o^ and 90^o^; (b) Fractured samples after flexural testing at different raster angles.

The stress–strain curves shown in Fig 9a indicate that the raster angle has a significant influence on the material behavior and fracture orientation of the samples during flexural testing (Fig 9b). The sample printed with a raster angle of 0° exhibited more brittle behavior, whereas the samples with 45° and 90° raster angles exhibited more ductile behavior. The sample with a 0^o^ raster angle exhibits minimal resistance to bending because the interraster bond, which is relatively weaker than the in-raster bond, allows the fracture to propagate along the raster deposition. In a 0° raster orientation, the bending resistance of a sample depends only on the bonds between rasters and not on the strength of the carbon fibers. Thus, sample fracture is caused by the delamination mechanism between the rasters. A similar observation in the tensile test of FDM printed carbon fiber reinforced PA samples was also reported in [26]. For samples with 45^o^ and 90^o^ raster angles, the fracture starts on the tension side, whereas on the compression side, the undamaged rasters keep the fracture elements together. In the 90^o^ raster orientation, the fracture propagated almost linearly along the applied load, with a slight zigzag depending on the in-raster failure. In this case, the bending resistance of the samples mostly depends on the adhesive bond between the carbon fibers and the polymer matrix, that is, it depends on the quality of the in-raster bond. A similar effect of a 90^o^ raster angle on the fracture orientation of FDM printed PLA samples was demonstrated [45]. The samples with 45^o^ raster angle exhibited the highest ductility. The reason for this is the fracture deflection during raster deposition. Once a fracture is directed away from the highest tensile stresses, further fracture propagation demands more energy, resulting in greater ductility.

**Fig 10:**
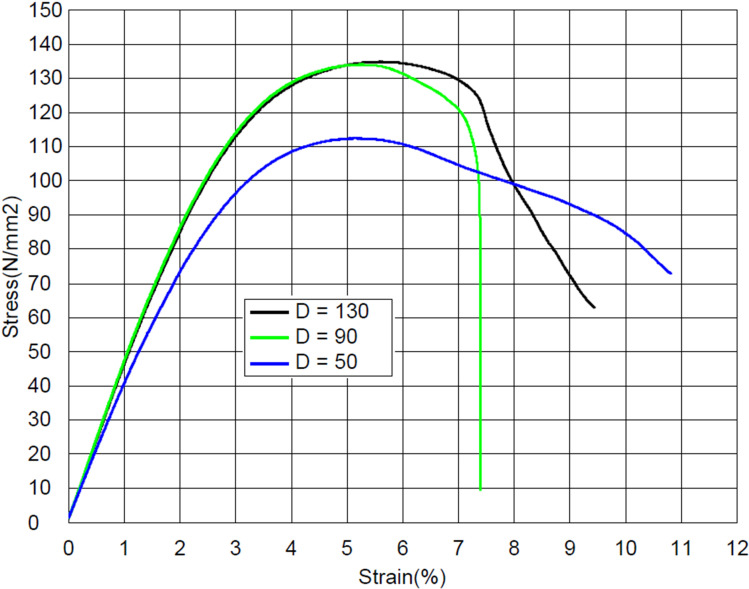
The stress–strain curves at different build plate temperatures.

At a higher build plate temperature, a stronger bond between individual layers was achieved, that results in higher flexural strength of samples (Fig 10). Although at a lower build plate temperature, the samples exhibited more ductile behavior, which can be explained by the fact that weaker bond between individual layers allows the material to deform more before breaking.

**Fig 11:**
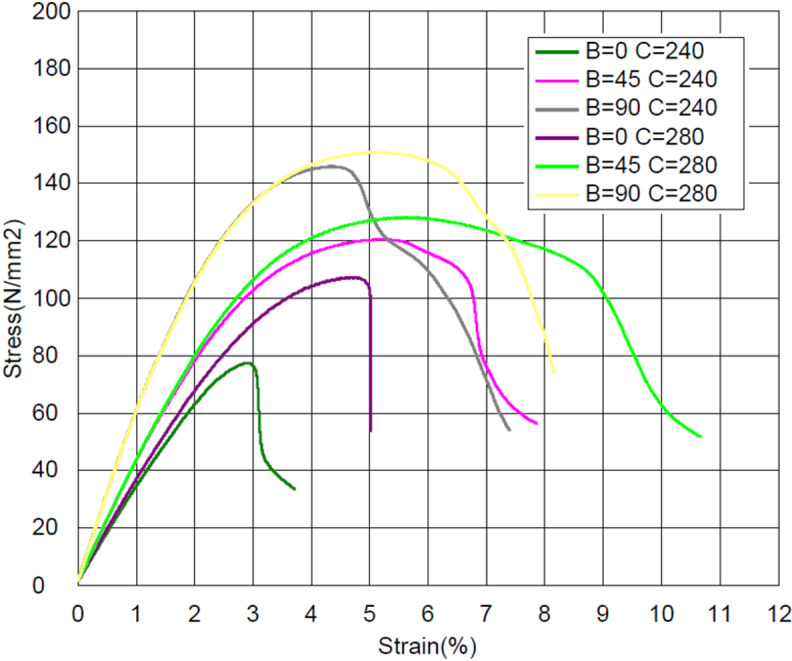
The stress–strain curves at different raster angles and printing temperatures.

The printing temperature is an important process parameter in the FDM process [46,47], as shown in Fig 11. At a higher printing temperature for all three raster angles (0^o^, 45^o^ and 90^o^), a higher flexural strength was achieved, and the material was more ductile. The reason for this is that increasing the printing temperature results in stronger interlayer bonds and interraster bonds, as well as stronger bonds between the carbon fibers and the polymer matrix [18,19,48]. Overall, the optimal selection of the process parameters led to a significant improvement in the flexural properties of the FDM printed carbon fiber reinforced polyamide material.

### 4.3. ANN optimization

**Fig 12:**
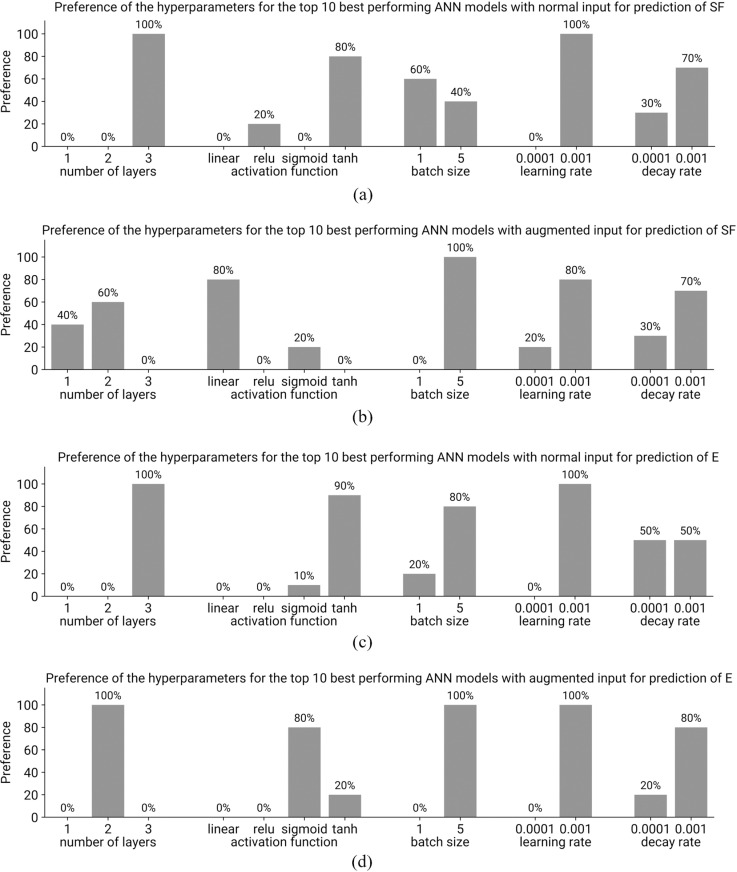
Preferences in the hyperparameters used in ANN models training for the top ten best performing models.

Using the proposed ANN approach, 100 configurations of each ANN model (with normal and augmented inputs) were generated. The top ten best-performing models were then selected from the ranking list to determine the optimal ANN architecture and hyperparameters. The plots in Fig 12a–12d illustrate the preference for hyperparameters in these top-performing models for predicting SF and E. For models with normal input (Figs 12a and 12c), architectures with 3 layers are optimal. For models with augmented input (Fig 12b and 12d), 2-layer architectures are generally optimal, although a single-layer architecture may suffice for predicting SF. The optimal activation function for models with normal input is tanh. For models with augmented input, the linear and sigmoid activation functions are optimal. A batch size of 5 is optimal for augmented input models, whereas batch sizes of 1 and 5 are both suitable for models with normal input. A learning rate of 0.001 is optimal across all the models. While both decay rates were present among the best-performing models, there was a clear preference for a decay rate of 0.001. On the basis of the performance of these models, the optimal hyperparameter values for ANN models with normal and augmented inputs for predicting SF and E are shown in Table 9.

**Table 9:** Optimal hyperparameter values of ANN models.

Flexural property	Input	Number of layers	Activation function	Batch size	Learning rate	Decay rate
SF	Normal	3	tanh	1	0.001	0.001
	Augmented	2	linear	5	0.001	0.001
E	Normal	3	tanh	5	0.001	0.0001/0.001
	Augmented	2	sigmoid	5	0.001	0.001

#### 4.3.1. ANN prediction of flexural strength and modulus.

**Fig 13:**
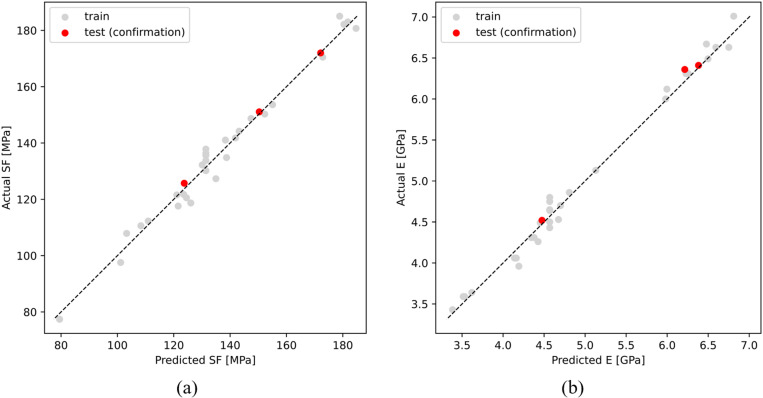
Plots of actual (experimental) vs. predicted values of (a) SF and (b) E.

To evaluate the accuracy and performance of the optimal ANN prediction models, new samples were 3D printed with a new set of process parameters and tested in a flexural test as a confirmation analysis of unseen data. The experimental data for three confirmation runs with randomly selected process parameters and the corresponding values of SF and E are shown in Table 10. The obtained performance metrics of these models are shown in Table 11. The results demonstrate that the proposed procedure effectively selects the best performing models, which achieve excellent outcomes on unseen data considering all the performance metrics. The first layer has the largest number of neurons, approaching the number of terms present in the second order model defined by (1), whereas the subsequent layers have significantly fewer neurons. This reduction in neuron count can be interpreted as a bottleneck, encouraging the second layer to focus on extracting the most important features. Compared with models with normal inputs, models with augmented inputs achieved substantially better performance, with lower error values and higher R^2^ values. This confirms the effectiveness of the optimization model proposed in this study, where unseen data were accurately predicted by the ANN for both flexural properties. To further analyze the ANN models, plots of actual versus predicted values for both SF and E are shown in Fig 13. These plots illustrate the relationship between the actual (experimental) and predicted values, with the dashed line representing the ideal correlation. The gray data points correspond to the values obtained during model training on the experimental data, whereas the red points represent the values obtained during model testing on the confirmation data. The symmetrical and closely distributed data points around the ideal line indicate that the models achieved highly accurate and unbiased predictions of SF and E for both experimental and unseen confirmation data.

**Table 10:** Confirmation dataset.

Exp. No.	Process parameters				Responses (average of three samples)	
	A	B	C	D	SF (MPa)	E (GPa)
1	0.8	90	280	90	171.99	6.41
2	1.4	90	260	50	151.07	6.36
3	2	45	280	50	125.71	4.52

**Table 11:** Performance metrics of the best performing ANN models with normal and augmented inputs for the prediction of SF and E on the confirmation dataset.

Flexural property	Input	ANN model configuration	MSE	RMSE	MAE	MAPE	R^2^
SF	Normal	Neurons per layer: (14, 3, 9); Activation function: tanh; Batch size: 5; Learning rate: 0.001; Decay rate: 0.001;	11.162	3.341	3.110	2.142%	0.969
	Augmented	Neurons per layer: (13, 3); Activation function: linear; Batch size: 5; Learning rate: 0.001; Decay rate: 0.001;	1.502	1.226	0.952	0.715%	0.996
E	Normal	Neurons per layer: (13, 2, 3); Activation function: tanh; Batch size: 5; Learning rate: 0.001; Decay rate: 0.001;	0.044	0.210	0.194	3.696%	0.943
	Augmented	Neurons per layer: (13, 6); Activation function: sigmoid; Batch size: 5; Learning rate: 0.001; Decay rate: 0.0001;	0.008	0.089	0.073	1.243%	0.989

#### 4.3.2. ANN models interpretability using SHAP.

**Fig 14:**
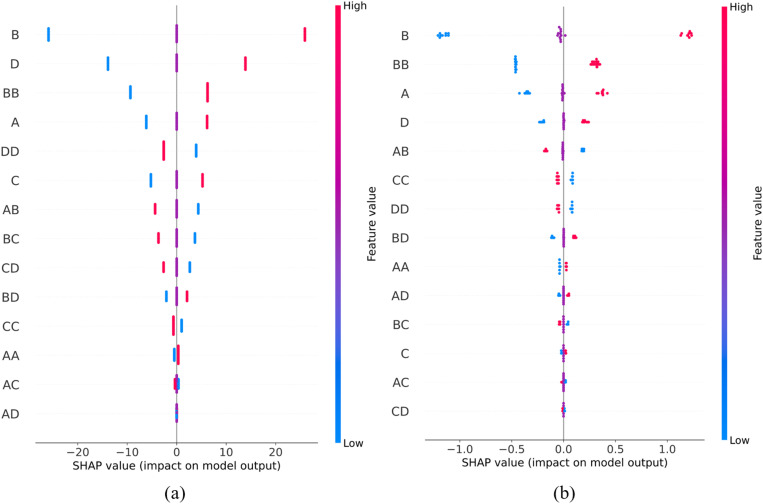
SHAP values of ANN models for predicting (a) SF and (b) E.

Fig 14 displays SHAP values of model features along with their relative importance and their relationships with the models’ outputs, i.e., predictions of SF and E. The left vertical axis represents the model features (variables), ranked by their importance, while the right vertical axis displays the corresponding feature values. A color gradient displays variable values, ranging from blue (low values) to red (high values). The x-axis represents SHAP values, which measure both the magnitude and direction of a variable’s influence on the model’s prediction. Positive SHAP values for a given variable indicate an increase in the predicted SF and E, while negative SHAP values suggest a decreasing effect.

Fig 14a shows that variables B, D, BB, and A have a wider distribution of SHAP values, implying their high importance in predicting SF. In contrast, variables AA, AC, and AD have narrow SHAP distributions, indicating their minimal impact on the prediction. A similar pattern is observed in Fig 14b, which shows the effect of variables on predicting E. Specifically, variables B, BB, A, and D have a significant influence on the prediction of E, while variables C, AC, and CD minimal impact. These findings suggest that variable B, its quadratic effect BB, as well as variables A and D, have the highest importance in predicting both SF and E. Furthermore, lower values of these variables (blue color) are associated with decrease (negative SHAP values) and higher values (red color) with increase (positive SHAP values) in predicted values of SF and E.

The analysis of the variable’s influence on the model’s output reveals that the data points cluster around three distinct SHAP values. Fig 14a shows the formation of three well-defined groups, characterized by complete alignment among the points. Similarly, as shown in Fig 14b, the clustering pattern remains consistent with the identified three primary groups, however, some minor variations in the distribution of points are present. Firstly, all variables are evaluated at three levels, leading to SHAP values that are likely to cluster around these three distinct values. Secondly, according to (5), SHAP defines a local linear explanation model, which is a simplified representation that cannot fully approximate the complex nonlinear transformations present in neural networks. Therefore, the SHAP values of individual samples (represented by points) in the model for predicting E exhibit variations, as shown in Fig 14b. Contrary, the ANN model for predicting SF does not use non-linear activation functions and is essentially a linear model of augmented inputs. As a result, the SHAP local explanation model can fully and consistently describe its behavior, leading to the complete alignment of points as shown in Fig 14a.

The SHAP analysis of the ANN models reveals that the importance of variables aligns with the variable importance obtained from the RSM model, as presented in the ANOVA tables (Tables 6 and 8). This consistency between the RSM and ANN models confirms the significance of the identified key variables in predicting SF and E, and additionally it confirms adequacy of ANNs in predicting flexural properties of CFrPA material.

### 4.4. Comparison of RSM and ANN models

**Fig 15:**
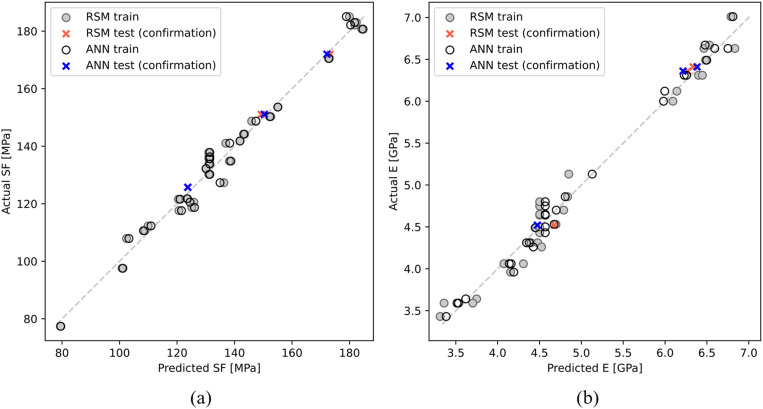
Actual vs. predicted plots of (a) SF and (b) E for RSM and ANN models.

The RSM models given by (7) and (8) and the ANN models with augmented inputs (Table 11) are compared in terms of MSE, RMSE, MAE, MAPE, and R^2^ for both SF and E. The performance metrics are presented in Table 12. Based on these metrics, it is evident that the ANN models with augmented inputs perform better in predicting both SF and E than the RSM models do, particularly in terms of deviation metrics (MSE, RMSE, MAE, and MAPE). The performance of the RSM and ANN models is examined by plotting the actual versus predicted values for both SF and E, as shown in Fig 15. These plots indicate that both the RSM and ANN models achieved a close and symmetrical distribution of data points around the ideal line, demonstrating high agreement between the experimental and predicted data in the confirmation test. The high accuracy of both models in predicting unseen data confirms their adequacy in predicting the flexural properties of polyamide reinforced with 10% short carbon fibers.

**Table 12:** Performance metrics of RSM models and best performing ANN models on the confirmation dataset.

Flexural property	Model	MSE	RMSE	MAE	MAPE	R2
SF	RSM	2.755	1.660	1.638	1.134%	0.992
	ANN	1.502	1.226	0.952	0.715%	0.996
E	RSM	0.013	0.114	0.108	2.029%	0.983
	ANN	0.008	0.089	0.073	1.243%	0.989

## 5. Conclusion

In this study, the impact of FDM process parameters on the flexural strength and modulus of polyamide reinforced with 10% short carbon fibers was experimentally investigated and predicted. Two prediction models, the RSM and ANN, were developed to predict flexural properties. Furthermore, ANN models were optimized to improve their predictive accuracy and reliability. The results demonstrate that these models can effectively predict the flexural strength and modulus of the CFrPA FDM-printed parts. The key conclusions from the research are as follows:

- The raster angle significantly affects flexural properties, with the build plate temperature having the next most significant impact. The printing temperature and wall thickness have a relatively small effect.
- The flexural strength and modulus of the CFrPA composite FDM-printed samples are the highest at a 90° raster angle and decrease as the raster angle is reduced.
- The samples printed with a 0° raster angle exhibit more brittle behavior, whereas those with 45° and 90° raster angles show more ductile behavior. At 0°, fractures propagate along the interraster bonds, whereas at 90°, fractures follow a zigzag pattern depending on in-raster failure.
- The optimized RSM and ANN models, characterized by low MSE, RMSE, MAE, and MAPE values and high R^2^ values, are effective for predicting the flexural strength and modulus of the CFrPA material.
- ANN models with augmented input achieved better predictive performance for both SF and E compared to RSM and ANN models with normal input. Additionally, ANN models with augmented input have a simpler architecture with two hidden layers, while models with normal input require three hidden layers.
