# Correction: Experimental investigation and prediction of the flexural properties of FDM printed carbon fiber reinforced polyamide parts using optimized RSM and ANN models

**DOI:** 10.1371/journal.pone.0333320

**Published:** 2025-09-25

**Authors:** The PLOS One Staff

The following information is missing from the Acknowledgement section: Researchers Supporting Project number (RSP2025R299), King Saud University, Riyadh, Saudi Arabia.

The publisher apologizes for the error.
